# Genome Analysis of *Acinetobacter lwoffii* Strains Isolated from Permafrost Soils Aged from 15 Thousand to 1.8 Million Years Revealed Their Close Relationships with Present-Day Environmental and Clinical Isolates

**DOI:** 10.3390/biology10090871

**Published:** 2021-09-04

**Authors:** Andrey L. Rakitin, Alexandra Y. Ermakova, Alexey V. Beletsky, Mayya Petrova, Andrey V. Mardanov, Nikolai V. Ravin

**Affiliations:** 1 Research Center of Biotechnology of the Russian Academy of Sciences, Institute of Bioengineering, 119071 Moscow, Russia; rakitin@biengi.ac.ru (A.L.R.); alex.ermakova@mail.ru (A.Y.E.); mortu@yandex.ru (A.V.B.); mardanov@biengi.ac.ru (A.V.M.); 2 Institute of Molecular Genetics of National Research Centre “Kurchatov Institute”, 123098 Moscow, Russia; petrova@img.ras.ru

**Keywords:** *Acinetobacter lwoffii*, permafrost, genome, evolution, antibiotics resistance, plasmid, horizontal gene transfer

## Abstract

**Simple Summary:**

Arctic ecosystems are an extreme habitat characterized by a negative average annual temperature and the presence of permafrost that occupies about 25% of the land. Permafrost can retain viable microorganisms for several million years and therefore it is a source of unique “ancient” microbes. *Acinetobacter lwoffii* are aerobic chemoorganotrophic bacteria widespread in a variety of natural and artificial environments, and have been reported as hospital pathogens associated with nosocomial infections. Here, we carried out a genome-wide analysis of five strains of *A. lwoffii* isolated from permafrost aged from 15 thousand to 1.8 million years. Surprisingly, we did not reveal genetic determinants that distinguish them from modern clinical and environmental *A. lwoffii* isolates. On the phylogenetic tree permafrost strains do not form a separate cluster, but are related to various clinical isolates. The genomes of clinical and permafrost strains contain similar mobile elements and prophages, which indicates an intense horizontal gene transfer. Like clinical isolates, permafrost strains harbored antibiotic resistance genes, although plasmids from the modern strains are enriched with antibiotic resistance genes compared to permafrost ones. The obtained results indicate that thawing of permafrost caused by global warming could release new potentially pathogenic strains of *Acinetobacter* into the modern biosphere.

**Abstract:**

Microbial life can be supported at subzero temperatures in permafrost up to several million years old. Genome analysis of strains isolated from permafrost provides a unique opportunity to study microorganisms that have not previously come into contact with the human population. *Acinetobacter lwoffii* is a typical soil bacterium that has been increasingly reported as hospital pathogens associated with bacteremia. In order to identify the specific genetic characteristics of ancient permafrost-conserved strains of *A. lwoffii* and their differences from present-day clinical isolates, we carried out a genome-wide analysis of five strains of *A. lwoffii* isolated from permafrost aged from 15 thousand to 1.8 million years. Surprisingly, we did not identify chromosomal genetic determinants that distinguish permafrost strains from clinical *A. lwoffii* isolates and strains from other natural habitats. Phylogenetic analysis based on whole genome sequences showed that permafrost strains do not form a separate cluster and some of them are most closely related to clinical isolates. The genomes of clinical and permafrost strains contain similar mobile elements and prophages, which indicates an intense horizontal transfer of genetic material. Comparison of plasmids of modern and permafrost strains showed that plasmids from the modern strains are enriched with antibiotic resistance genes, while the content of genes for resistance to heavy metals and arsenic is nearly the same. The thawing of permafrost caused by global warming could release new potentially pathogenic strains of *Acinetobacter*.

## 1. Introduction

Arctic ecosystems are an extreme habitat characterized by a negative average annual temperature and the presence of permafrost soils. Permafrost is the part of the Earth’s upper crust that does not thaw when temperatures rise seasonally. Permafrost occupies about 25% of the land [1]. Russia ranks first in the world in terms of permafrost area, which makes up about 65% of its territory [2]. Permafrost soils contain a wide range of microorganisms including bacteria, archaea, algae, and fungi [3,4,5,6,7]. Permafrost can retain viable microorganisms under stable conditions at low temperatures for several million years [7,8] and therefore it is a source of unique “ancient” microorganisms.

Antibiotic resistance is a major global health problem that threatens to negate the benefits of the discovery of antibiotics [9]. Although genes for antibiotic resistance are common in natural microbial populations, a number of questions related to the role of human activity in the wide distribution of diverse determinants of resistance in pathogenic bacteria remain open [10,11]. In this regard, the search for antibiotic resistance genes in microorganisms that have not previously come into contact with the human population is of great interest [12]. Many studies on permafrost have reported the presence of genes coding for resistance to several classes of antibiotics [13,14,15,16,17,18] and heavy metals [19,20] similar to ones observed in present day clinical and environmental strains. The *Psychrobacter maritimus* strain, resistant to tetracycline and streptomycin, was isolated from 15,000–35,000 years old permafrost sediments sampled from the coast of the East Siberian Sea. The genes conferring resistance to streptomycin (*strA-strB*) and tetracycline (*tetR-tetH*) were localized on the plasmid pKLH80 [13]. As a result of the analysis of the genome of *Staphylococcus hominis* MMP2, isolated from 3.5 million years old permafrost soils in the Lena River area, genes encoding resistance to aminoglycosides, beta-lactams, macrolides and chloramphenicol were identified [8]. Perron et al. used functional metagenomics to retrieve antibiotic resistance genes from bacteria isolated from Canadian high Arctic permafrost and identified genes conferring clinical levels of resistance against aminoglycoside, β-lactam and tetracycline antibiotics [17]. Twenty eight bacterial species were cultured form a Siberian permafrost sample dated at 2.7 million years and resistance to antibiotics was phenotypically detected in all 10 Gram-negative species [18]. No significant differences in antibiotic resistance profiles between modern and ancient isolates of each species were found [18]. Direct evidence for the presence of antibiotic resistance genes in the permafrost microbiome has been obtained as a result of metagenomic analyses of 30,000-year-old permafrost sediments in Alaska [15]. This study revealed a highly diverse genes encoding resistance to β-lactam, tetracycline and glycopeptide antibiotics [15].

Bacteria of the genus *Acinetobacter*, Gram-negative cocci, belong to the family *Moraxellaceae*, order *Pseudomonadales*, class γ-Proteobacteria. *Acinetobacter* species are aerobic chemoorganotrophic saprophytes; they do not form spores, are not able to ferment glucose and carbohydrates. *Acinetobacter sp.* are widespread in various ecological niches [21,22,23]. *A. lwoffii* strains occur in a variety of natural and artificial environments such as forest and agricultural soils, animal and human skin and gut, fresh and seawater [24]. While *A. baumannii* is the most clinically important *Acinetobacter* species causing infections, *A. lwoffii* have been increasingly reported as hospital pathogens associated with nosocomial infections like septicemia, pneumonia, meningitis, urinary tract infections, skin, gastroenteritis and wound infections [25,26,27].

*Acinetobacter* sp. have a fairly variable genome that contains various mobile genetic elements, such as IS elements, transposons, plasmids and bacteriophages, which are often associated with antibiotic resistance [28,29,30]. Until now, most studies on sequencing and genome analysis have been devoted to *A. lwoffii* strains isolated from clinical material. Only a few genomes of *A. lwoffii* strains isolated from natural environments have been reported, namely *A. lwoffii* ZS207 from a microbial mat in a gold mine [31], *A. lwoffii* M2a from a honey sample but probably originated from bee intestines [32], and *A. lwoffii* GC2 from petroleum-contaminated soil [33,34].

In order to identify the specific genetic characteristics of ancient permafrost-conserved strains of *A. lwoffii* and their differences from present-day clinical isolates, we carried out a genome-wide analysis of five strains of *A. lwoffii* isolated from permafrost aged from 15 thousand to 1.8 million years. Surprisingly, we did not reveal chromosomal genetic determinants that distinguish permafrost strains from clinical strains and strains from other non-clinical environments.

## 2. Materials and Methods

### 2.1. Strains of A. lwoffii

The objects of the study were five strains of *A. lwoffii* isolated from samples of permafrost sediments collected in the Kolyma lowland region, the Republic of Yakutia, Russia [35]. We herein referred to these strains as “permafrost” strains, which never come into contact with clinical isolates. It was previously shown that most of the studied strains are resistant to antibiotics and heavy metals [20,35], as summarized in Table 1.

All strains were grown in lysogeny broth (LB) medium or solidified LB medium (LA) at 30 °C. When required, antibiotics were added at the following final concentrations (μg mL^−1^): streptomycin (Sm) 100, spectinomycin (Sp) 100–200, chloramphenicol (Cm) 20, and ampicillin (Amp) 100.

### 2.2. Genome Sequencing and Assembly

A single colony of the *A. lwoffii* strain was inoculated with 5 mL of LB medium and grown with shaking at 30 °C for 16–20 h. Cells were harvested by centrifugation (10,000× *g* for 5 min), and total DNA was isolated using a DNeasy PowerSoil Kit (Qiagen). 

Genomic DNA was sequenced using Illumina and Oxford Nanopore platforms. The shotgun genome library was prepared using the NEBNext Ultra II DNA library prep kit (New England BioLabs, Ipswich, MA, USA). The obtained libraries were sequenced on Illumina MiSeq (Illumina, San Diego, CA) using MiSeq Reagent Kit v3 (600-cycle) in a paired reads (2 × 300 nt) mode (Supplementary Table S1). Adapter removal and trimming of low-quality sequences (Q < 30) were performed using Cutadapt v.1.8.3 [36] and Sickle v.1.33 (https://github.com/najoshi/sickle, accessed on 27 July 2021), respectively. Trimmed reads were merged using FLASH v.1.2.11 [37].

Genomic DNA was additionally sequenced on a MinION system (Oxford Nanopore, UK) using the 1D Genomic DNA by ligation protocol and kit (SQK-LSK108). The libraries were sequenced in an R9.4 flow cell (FLO-MIN106) using MinION device (Supplementary Table S1).

The resulting Illumina and Nanopore reads were *de novo* assembled into contigs using hybrid assembler Unicycler v. 0.4.8 [38].

### 2.3. Annotation and Analysis of the Genomes

Gene search and annotation of obtained genomes were performed using the RAST server 2.0 [39].

The core genome of *A. lwoffii* strains was determined by clustering all genes selected by the BLASTclust program from the BLAST package [40]. Genes whose predicted protein products had more than 50% amino acid sequence identity across more than 80% of the length were combined into one cluster. Clusters containing at least one protein from each genome formed the core genome. 

For genome-based phylogenetic analysis, 1585 single copy genes present in each genome (14 strains of *A. lwoffi* and *A. pseudolwoffii* CIP 64.10) were identified by clustering of all genes at 70% nucleotide sequence identity threshold, using the Blastclust program of the NCBI BLAST package. For each single copy gene multiple alignment was build using MAFFT program [41]. The obtained alignments were concatenated, and the poorly aligned regions were removed using Gblocks [42]. Based on the obtained alignment, a maximum likelihood tree was built using the PhyML [43] with default parameters (HKY85 nucleotide substitution model was used, equilibrium base frequencies were estimated by counting the occurrence of bases in the alignment, gamma distribution with estimated shape parameter was used to model 4 substitution rate categories, no invariant sites, branch support values were calculated using the approximate Bayes method).

The average nucleotide identity (ANI) between the genomes was calculated using the ani.rb script from the Enveomics Collection [44].

The copy numbers of plasmids relative to the chromosome was deduced from the average sequencing depths of the corresponding contigs calculated by Unicycler v. 0.4.8.

The Comprehensive Antibiotic Resistance Database (CARD) [45] was used to search for antibiotic resistance genes. The ISfinder server [46] was used to search for insertion sequences. The search for prophages was performed using the PHASTER program [47], followed by verification of the results by searches against NCBI databases. To identify composite transposons, we searched for pairs of IS elements (nucleotide sequence identity above 99%) located at a distance of up to 15 kb in the same orientation.

A concatenated amino acid sequence of eight proteins (UreABCDEFGJ) was used to construct a phylogenetic tree for the urease operon. The sequences were aligned using the MUSCLE algorithm in the MEGA package [48]; the phylogenetic tree was constructed using the maximum likelihood method [49].

### 2.4. Determination of the Ability of A. lwoffii Strains to Degrade Urea

For the analysis, a plate test on Christensen medium was used [50]. A single colony was inoculated into 5 mL of LB and grown overnight at 30 °C. An aliquot was taken, inoculated into 5 mL of Christensen medium with glucose and urea, and grown overnight. The OD_600_ of the culture was adjusted 0.15. Then, 3 μL of the resulting culture was dropped onto a Christensen Urea Agar plate. The cultures were grown for 24 h at 30 °C. Increase in pH due to the production of ammonia from urea hydrolysis results in a color change from yellow (pH 6.8) to bright pink (pH 8.2). 

## 3. Results and Discussion

### 3.1. General Genome Characteristics

The genomes of *A. lwoffii* strains ED23-35, ED45-23, ED9-5A, VS15 and EK30A were sequenced using a combination of Illumina and Nanopore technique. Following hybrid *de novo* assembly using the Unicycler assembly pipeline, a complete circular chromosome sequence and a set of contigs representing plasmids were obtained for each strain. The size of the chromosomes of the sequenced strains varied from 3.16 to 3.26 Mbp, the number of plasmids was from 6 to 15 (Table 2). 

All contigs were assembled as circular molecules and thus represented the complete chromosome and plasmid sequences. All plasmids, previously reported in strains ED23-35, ED45-23 and VS15, were identified in the assemblies with the exception of plasmids pALWED 1.6 (strain ED23-35) and pALWED 3.4 (strain ED9-5A), whose sequences were found in the chromosomes of the corresponding strains. In the EK30A strain plasmids pALWEK 1.6, pALWEK 1.9 and pALWEK 1.11 were not found, but one previously unreported plasmid, designated pALWEK 1.17 (4130 bp long), was discovered. One plasmid, pALWED 1.8, was found in strains ED23-35, VS15 and EK30A, while other plasmids occurred in one strain only.

The ANI values between all permafrost strains, as well as between permafrost and nine other strains of *A. lwoffii* were above the 95% threshold for species delineation [51,52], indicating that all of them belong to the same species (Supplementary Table S2).

Annotation of the genome sequences predicted from 3387 to 3547 protein-coding genes, among which about 70% were functionally annotated. Each genome contained 7 copies of 5S rRNA, 16S rRNA and 23S rRNA, and from 83 to 87 tRNA genes (Table 3).

### 3.2. Core Genomes and Phylogenetic Relationships of Environmental and Clinical Strains of A. lwoffii

For the pangenome analysis, we used nine genomes of *A. lwoffii* deposited in the GenBank database and five genomes of permafrost strains sequenced in this work. Genomes of *A. lwoffii* strains ZS207, M2a, GC2 and five permafrost strains were combined into the “environmental” group, six genomes of clinical *A. lwoffii* strains were allocated into the “clinical” group (Table 4). The core genome of environmental and clinical groups comprised 2224 and 2266 genes, respectively. The core genome of all 14 strains of *A. lwoffii* consisted of 2096 genes.

The core genome of environmental strains exceeds the total core genome by only 128 genes (Supplementary Table S3). Most of these genes were absent in only one (101 genes) or two (19 genes) out of 6 clinical strains; only one gene was present in all environmental isolates and absent in all analyzed clinical strains. It encodes a TauE/SafE family sulfite exporter. TauE/SafE proteins are involved in the transport of anions across the cytoplasmic membrane and were found to act as sulfite/organosulfonate exporters in the metabolism of C2 sulfonates [53]. In all permafrost strains two copies of these genes are located on the chromosomes and in strain ED45-23 an additional copy was found on plasmid pALWED2.1. Organosulfonates are widespread in nature, particularly in the humic material of soil [54], and probably could be utilized by permafrost strains.

A similar picture was observed when comparing the core genome of clinical strains with the core genome of all strains (Supplementary Table S4). We found 170 genes common to all clinical strains were absent in one or several (maximum five out of eight) environmental strains. The real number of differences between the core genomes of environmental and clinical strains is probably even smaller, since the observed differences are primarily associated with the quality of the genome assembly. For example, the complete genome of strain ED9-5A lacked only six genes present in all clinical isolates, while 62 genes were not identified in the draft genome of strain GC2, assembled into 285 contigs.

However, the absence of three genetic loci in environmental isolates should be noted. Two environmental strains, ZS207 and ED23-35, lacked genes encoding the copper resistance proteins CopC and CopD, while strains GC2 and ED23-35 lacked the complete biotin biosynthesis operon. Four permafrost strains lack the gene encoding competence protein ComEA involved in uptake of exogenous DNA [55]. Perhaps these functions are more in demand in the clinical environment. Particularly, natural competence is common among clinical isolates of *Acinetobacter* spp., and is an important trait for acquisition of antibiotic resistance [56]. Copper resistance may play a role in survival in the human host or hospital environment and is important for full virulence of *A. baumannii* [57].

**Figure 1:**
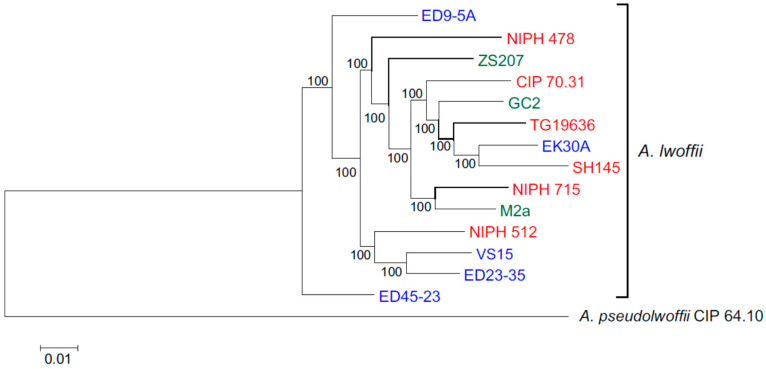
Genome-based phylogeny of *A. lwoffii*. The maximum likelihood tree is based on concatenated nucleotide sequences of 1585 single copy genes. Clinical, permafrost and other natural isolates of *A. lwoffii* are shown in red, blue and green, respectively. *A. pseudolwoffii* CIP 64.10 was used to root the tree.

Concatenated nucleotide sequences of 1585 single copy genes were used for phylogenetic analysis of 14 strains of *A. lwoffii*. The structure of the resulting tree showed that environmental and clinical strains do not form separate clusters (Figure 1). Moreover, five strains isolated from permafrost soils do not form a separate branch, and one of them, EK30A, clustered with the clinical strain SH145, with which it also has the highest ANI value. Only two permafrost strains, VS15 and ED23-35, turned out to be very close on the phylogenetic tree, and they also had the highest ANI of 97.64%.

### 3.3. Antibiotics Resistance Genes

As a result of a comparative analysis of the genomes of clinical and environmental strains of *A. lwoffii*, potential genes for antibiotic resistance that are common and unique for each group were identified (Table 5). All clinical and environmental strains contain genes encoding the carbapenem-hydrolyzing class D β-lactamase (*bla_OXA-134_*), chloramphenicol acetyltransferase (*cat*), and ABC family transport proteins MacAB-TolC. The *bla_OXA-134_* gene is widespread in *A. lwoffii* and has been proposed as a tool for rapid identification of this species [58]. All these common resistance genes are localized on the chromosomes.

Strains ED23-35, VS15, and EK30A are resistant to streptomycin and spectinomycin [35], which is determined by the *aadA27* gene located on the plasmid pALWED1.8 [16]. The VS15 strain is resistant to chloramphenicol [35] due to the presence of the *cflA* gene on the plasmid pALWVS1.4 [59]. The experimentally shown weak resistance of strain ED23-35 to tetracycline is probably determined by plasmid pALWED1.1 (GenBank KX426227) carrying the *tet(H)* gene (our unpublished data). Genes *aph*(3″)-Ib and *aph*(6)-I found in the strain TG19636 are also located on a plasmid.

Thus, it can be assumed that antibiotic resistance genes specific for individual strains are localized on plasmids that can facilitate their horizontal transfer. At the same time, genes of chloramphenicol acetyltransferase, β-lactamase, and MacAB-TolC efflux transporters common for clinical and environmental strains are located on the chromosome and have been vertically inherited. 

### 3.4. Mobile Elements

Insertion sequences play an important role in the evolution of the host genome and are involved in mutagenesis and gene activation, as well as in the rearrangement of chromosomes and plasmids [60]. IS elements are typically small (700–2500 bp) self-transferable genetic elements that contain usually one open reading frame flanked by inverted repeats and encoding a transposase that catalyzes DNA cleavage and transfer of the IS element [61].

The ISfinder database [46] contains 135 IS elements that were found in the genomes of various *Acinetobacter* species. IS elements in the genomes of permafrost strains of *A. lwoffii* were identified by searches against the ISfinder database [46]. For further analysis, only intact IS elements exhibiting more than 95% nucleotide sequence identity with the IS elements deposited in the database, were retained.

As a result, it was found that the genomes of permafrost strains contained from 70 to 133 copies of IS elements (Table 6 and Supplementary Table S5). The number of IS elements varied from 25 in strain ED23-35 to 39 in strain EK30A. Strains ED23-35 and ED45-23 contains 9 families of IS elements, three other permafrost strains harbored IS elements representing 11 families. Genomes of all environmental strains contain IS*1*, IS*3*, IS*4*, IS*5*, IS*6*, IS*30*, IS*982*, IS*NCY* families. The genome of strain ED9-5A contains IS*21* and IS*L3*, which are absent in four other permafrost strains. The genome of *A. lwoffii* ZS207 additionally contains the IS*21*, IS*200*, IS*481*, IS*701* and IS*L3* families, and the genome of *A. lwoffii* M2a contains IS*21*, IS*701* and IS*1595*. The genome of the strain M2a contains the largest number of different IS elements. In general, in terms of the number and diversity of IS elements, permafrost strains do not differ significantly from the other environmental strains.

Most of the IS elements in the genomes of environmental strains are present in 1 to 10 copies per genome. The number of copies of IS*Aba11* of the IS*701* family, found in the genomes of *A. lwoffii* ED23-35, VS15, EK30A, and M2a, ranges from 8 to 13; however, the genome of strain ZS207 contains only two copies of IS*Aba11*, the genome of strain GC2 contains only one copy, and the strains ED45-23 and ED9-5A do not contain this IS element (Supplementary Table S5).

Analysis of the presence of IS elements in clinical strains of *A. lwoffii* was more complicated because all these genomes were assembled at the scaffold level (from 9 to 245). The actual copy number of IS elements could be underestimated due to collapsing of multiple identical IS copies into a single contig, that is particularly evident for the draft genome of strain TG19636. Nevertheless, both SH145 and NIPH 478 clinical strains were shown to contain the least variety of IS families (Table 7).

Analysis of genomes of environmental strains of *A. lwoffii* revealed the presence of several composite transposons, which are mobile genetic elements consisting of two IS elements flanking a DNA fragment often containing antibiotic resistance and other adaptive genes. Putative composite transposons were found in four out of five permafrost strains and in strain M2a but in none of the clinical isolate (Table 6 and Table 7). Only one transposon, found in strain VS15, carried antibiotic resistance determinant, while most other harbored genes involved in various metabolic processes (Supplementary Table S6). Two of the composite transposons found in permafrost strains were also found in clinical *A. lwoffii* strains FDAARGOS 1393 and FDAARGOS_552 (Supplementary Table S6).

Earlier, based on the analysis of more than 200 bacterial genomes, it was shown that composite transposons, mostly containing antibiotic resistance genes, are most often found in clinical bacterial strains, usually on plasmids [62]. Wagner suggested that the preservation of spontaneously occurring composite transposons is stimulated by strong selective pressure in the clinic [62]. Therefore, the presence of a larger number of composite transposons in environmental *A. lwoffii* strains compared to clinical ones is unexpected.

### 3.5. Plasmids

Since the comparison of the complete genomes of *A. lwoffii* strains did not reveal significant differences between clinical and environmental, or modern and permafrost isolates, we decided to compare the plasmids of modern and permafrost strains. As mentioned above, the genomes of modern strains that we used for comparison, with the exception of ZS207, were not assembled at the level of complete chromosomes and plasmids. Therefore, we compared the frequency of occurrence of genes for resistance to heavy metals and antibiotics among permafrost and modern plasmids of *A. lwoffii*. 

Among 41 permafrost plasmids, only three (7.3%) harbored one antibiotic resistance gene, and seven plasmids (17.1%) contained resistance genes to various heavy metals and arsenic (Supplementary Table S7). Among the 64 modern plasmids isolated from various sources, eight (12.5%) contained from one to three antibiotic resistance genes and 11 (17.2%) plasmid carrying genes for resistance to heavy metals and arsenic. Thus, it is obvious that modern plasmids are enriched with antibiotic resistance genes compared to permafrost ones, while the content of genes for resistance to heavy metals and arsenic in both samples are comparable. It should be noted that *A. lwoffii* strains, unlike *A. baumanii*, are part of the normal microbiota of the skin and mucous membranes of healthy people [63], so most strains isolated from human samples can hardly be considered pathogenic. However, modern strains of *A. lwoffii*, when exposed to antibiotics, begin to accumulate appropriate resistance genes, which is typical for clinical and veterinary samples. The accumulation of new adaptive genes occurs so far only on plasmids, and large plasmids (>40 kb) play a major role in such adaptation.

### 3.6. Integrated Phages

To date, more than 100 bacteriophages infecting bacteria of the genus *Acinetobacter* have been described. They belong to the families Siphoviridae, Podoviridae, and Myoviridae [64]. As a result of the analysis of the genomes using the PHASTER program, 52 prophages integrated into the chromosome were found, of which 11 were presumably intact. The sizes of intact prophages ranged from 18.9 to 50.4 kb (Table 8).

Although no completely identical prophages were found in different permafrost strains, many prophages contained extended regions (up to 55% of their length), that had a high (>90%) nucleotide sequence similarity to intact prophages of other strains (Table 8). Probably this is a result of active engagement of horizontal genetic exchange in evolution of *Acinetobacter* phages leading to formation of phage genomes mosaic in their architectures [65].

The sequences of 11 intact prophages from permafrost strains were compared with the genomes of *A. lwoffii* strains deposited in GenBank. For two prophages, similar sequences (more than 70% identity over 70% of the prophage length) were found in the genomes of various clinical strains of *A. lwoffii*. A region similar to ED45-23-1 prophage was found in the genome of the *Acinetobacter johnsonii* E10B (89% identity over 85% length) from prawn digestive tract. Near complete (99% identity over 94% length) copy of EK30A-8 prophage was identified in the genome of clinical strain *A. lwoffii* FDAARGOS_551.

### 3.7. Urea Utilization Operon and its Functional Activity in Permafrost Strains

The presence of genetic determinants of urea utilization is one of the features that distinguish environmental strains of *A. lwoffii* from clinical isolates. The urease operon enabling the hydrolysis of urea into carbon dioxide and ammonia was identified in the genomes of all five permafrost strains of *A. lwoffii*, as well as in strains M2a and GC2, but was absent only in the genome environmental strain ZS207. On the contrary, of the six analysed clinical strains of *A. lwoffii*, only two contained the urease operon.

The operon includes the *ureABC* genes encoding the three subunits of urease and the auxiliary genes *ureEFGD* encoding the proteins required for the synthesis of the catalytically active Ni-containing form of the enzyme [66]. The product of another gene, *ureJ*, belongs to the pf04955 family of translocases and is likely to transport nickel into the cell [67].

**Figure 2:**
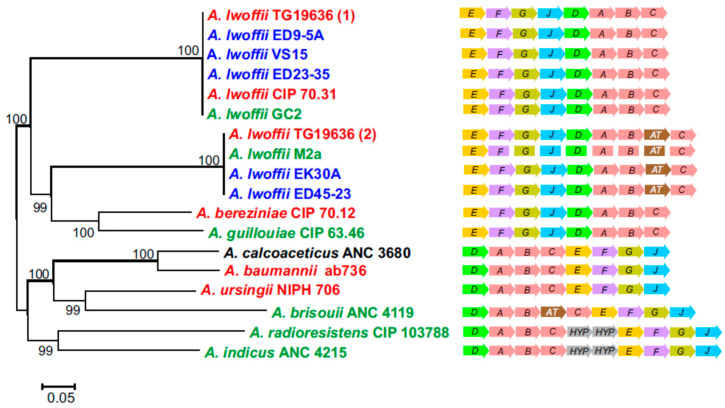
Structural organization and phylogeny of urease operon in *Acinetobacter*. The maximum likelihood phylogenetic tree was based on the concatenated amino acid sequences of eight proteins of the urease operon. Clinical, permafrost and other natural isolates of *Acinetobacter* are shown in red, blue and green, respectively. The origin of *A. calcoaceticus* AMC 3860 is unknown. AT, N-acetyltransferase gene; HYP, genes encoding hypothetical proteins.

The structures of urease operons in clinical and environmental strains of *A. lwoffii* are similar (Figure 2). In strains ED45-23 and EK30A, which have identical structures of the urease operon, the N-acetyltransferase gene (pfam13673) is inserted between the *ureB* and *ureC* genes. An operon with identical structure was found in the environmental strain M2a. In the clinical strain TG19636 there are two copies of the urease operon, one of which has the standard *ureABC* structure, and the second additionally contains the N-acetyltransferase gene (Figure 2). Similar structure of urease operons (without the N-acetyltransferase gene) was observed in *A. bereziniae* and *A. guillouiae*, while in other *Acinetobacter* species the order of genes is different (Figure 2).

Phylogenetic analysis of concatenated amino acid sequences of proteins (UreA, UreB, UreC, UreD, UreE, UreF, UreG, and UreJ) of the urease operons revealed clustering of two structural types of the operons in *A. lwoffii*, rather than operons of environmental and clinical strains of this species (Figure 2).

In addition to the urease operon in the genomes of all analysed environmental and clinical strains, the *urtABCDE* operon, which encodes the urea ABC transporter induced under nitrogen deficiency [68], was identified. In all these strains the *urt* operon is located on the chromosome. Urea imported into the cell can be cleaved either by urease or by a pair of enzymes, urea carboxylase and allophanate hydrolase. Genes encoding both enzymes are present in all analysed *A. lwoffii* strains.

**Figure 3:**
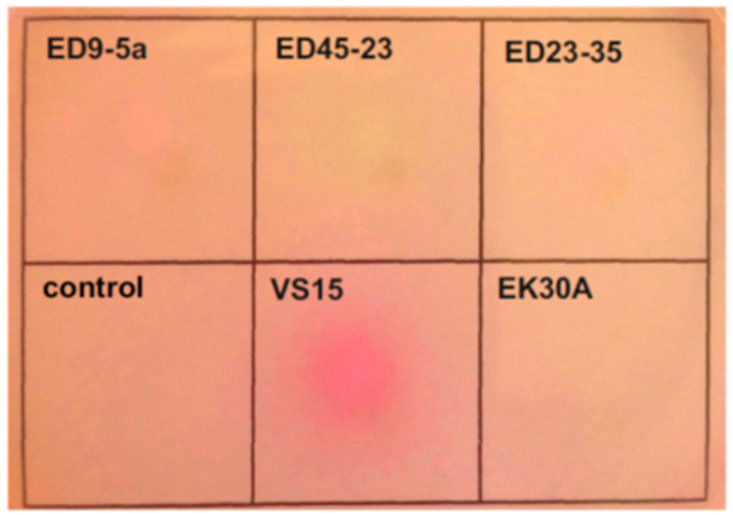
Evaluation of urase activity by Christensen’s method. Cultures were dropped onto a Christensen Urea Agar plate and grown overnight. Christensen liquid medium was used as a negative control.

The ability of the permafrost strains to utilize urea as a nitrogen source was determined by inoculation on Christensen’s agar [50]. A change in the colour of the medium, reflecting a change in pH due to the hydrolysis of urea, was reliably detected only in the case of strain VS15 (Figure 3).

In *A. lwoffii* strains ED23-35 and VS15, the urease operon is localized on plasmids pALWED1.2 and pALWVS1.1, respectively, while in strains ED45-23, ED9-5A, and EK30A, this operon is located on the chromosome. In contrast to pALWED1.2, the copy number of which is about one per chromosome, the copy number of pALWVS1.1 is higher (about 3.6), which probably leads to higher urease activity due to the gene dose effect.

## 4. Conclusions

Analysis of genomes of five *A. lwoffii* strains isolated from permafrost aged from 15 thousand to 1.6 million years did not reveal genetic determinants that distinguish them from clinical *A. lwoffii* strains and strains from other non-clinical environments. Phylogenetic analysis based on whole genome sequences also showed that permafrost strains do not form a separate cluster, but are related to various clinical isolates. The genomes of clinical and permafrost strains contain similar mobile elements and prophages, which indicates an intense horizontal transfer of genetic material. However, comparison of plasmids of modern and permafrost strains showed that modern plasmids are enriched with antibiotic resistance genes, while the content of genes for resistance to heavy metals and arsenic is nearly the same. The thawing of permafrost caused by global warming could release new strains of *Acinetobacter* that could be potentially pathogenic to humans.

## Figures and Tables

**Table 1:** Analyzed permafrost strains of *A. lwoffii*.

Strain	Isolation Depth (m)	Age of Permafrost (Thousand Years)	Resistance to Antibiotics	Resistance to Heavy Metals
ED23-35	4.5	20–40	Sm, Sp	Hg, Cr, Co, Cd, Zn, Ni
ED45-23	2.9	20–40	-	Hg, As, Cu
ED9-5A	6.5	15–30	-	Hg, As, Cr, Cd, Zn, Cu
VS15	34.0	20–40	Amp, Cm, Sm, Sp	Co, Cd, Zn, Cu
EK30A	47.9	1600–1800	Amp, Sm, Sp	Cr, Co, Cd, Cu

**Table 2:** Genomes of permafrost strains of *A. lwoffii*.

Strain	Chromosome	Plasmids		
	Size (bp)	Name	Size (bp)	Resistance Genes ^1^
ED23-35	3,160,760	pALWED 1.1	287,861	*tet(H)*, *mer*, *chr*, *czc*, *nreB*
		pALWED 1.2	48,955	
		pALWED 1.3	16,067	*chr*
		pALWED 1.4	14,116	
		pALWED 1.5	6715	
		pALWED 1.7	4861	
		pALWED 1.8	4135	*aadA27*
ED45-23	3,260,192	pALWED 2.1	196,557	*mer*, *ars*, *cop*
		pALWED 2.2	43,270	
		pALWED 2.3	22,769	
		pALWED 2.4	11,092	
		pALWED 2.5	10,584	
		pALWED 2.6	9201	
		pALWED 2.7	8816	
		pALWED 2.8	8120	
		pALWED 2.9	6308	
ED9-5A	3,231,133	pALWED 3.6	185,756	*mer*, *ars*, *cop*, *czc*
		pALWED 3.1	138,030	
		pALWED 3.5	16,567	*chr*
		pALWED 3.2	15,656	
		pALWED 3.7	9958	
		pALWED 3.3	8055	
VS15	3,260,140	pALWVS 1.1	134,096	*cop*, *czc*
		pALWVS 1.2	15,780	
		pALWVS 1.4	11,964	
		pALWVS 1.3	10,985	
		pALWVS 1.5	4677	
		pALWED 1.8	4135	*aadA27*
EK30A	3,183,510	pALWEK 1.1	209,982	
		pALWEK 1.2	12,172	
		pALWEK 1.12	11,382	
		pALWEK 1.3	10,347	
		pALWEK 1.10	9202	
		pALWEK 1.13	8910	
		pALWEK 1.4	8635	*cflA*
		pALWEK 1.5	8227	*chr*
		pALWEK 1.7	6691	
		pALWEK 1.8	5324	
		pALWEK 1.14	4760	
		pALWEK 1.15	4677	
		pALWED 1.8	4135	*aadA27*
		pALWEK 1.17	4130	
		pALWEK 1.16	2621	

^1^ The *tetR(H)* gene confers resistance to tetracycline, *cflA*—to chloramphenicol/fluorochloramphenicol, *aadA27*—to streptomycin/spectinomycin, *nreB*—to Ni, *czc*—to Co/Zn/Cd, *cop*—to Cu, *chr*—to Cr, *ars*—to As, *mer*—to Hg.

**Table 3:** General characteristics of the genomes.

Parameter	Strain				
	ED23-35	ED45-23	ED9-5A	VS15	EK30A
Predicted genes	3560	3644	3653	3496	3495
Protein-coding genes	3453	3537	3547	3392	3387
Protein-coding genes with predicted function	2362 (68,4%)	2463 (69.6%)	2543 (71.7%)	2404 (70.9%)	2405 (71.0%)
tRNA genes	86	86	85	83	87
G + C content (chromosome)	43.22	43.20	43.21	43.26	43.01

**Table 4:** Core genomes of clinical and environmental strains of *A. lwoffii*.

Strain	Source	Protein-Coding Genes	Genes in the Core Genome
**Environmental group**			**2224**
ED23-35	Permafrost	3453	
ED45-23	Permafrost	3537	
ED9-5A	Permafrost	3547	
VS15	Permafrost	3392	
EK30A	Permafrost	3387	
ZS207	Gold mine	3230	
M2a	Honey	3533	
GC2	Petroleum-contaminated soil	3267	
**Clinical group**			**2266**
SH145	Skin	3134	
NIPH 715	Pus	3314	
CIP 70.31	Gangrenous lesion	3503	
NIPH 478	Ear swab	3088	
NIPH 512	Unknown	3237	
TG19636	Urine	3412	

**Table 5:** Antibiotics resistance genes in the *A. lwoffii* genomes.

Gene	Protein	Strain Group	
		Clinical (6)	Environmental (8)
*cat*	Chloramphenicol acetyltransferase	6	8
*bla_OXA-134_*	OXA-134 family class D β-lactamase	6	8
*macAB*, *tolC*	Drug efflux ABC-type transporter	6	8
*sul*2	Dihydropteroate synthase	3 ^1^	0
*aph*(3″)-Ib	Aminoglycoside 3′-phosphotransferase	1 ^2^	0
*aph*(6)-I	Aminoglycoside-6-phosphotransferase	1 ^2^	0
*aadA27*	Streptomycin- spectinomycin 3″-adenylyltransferase	0	3
*tet(H)*	Tetracycline efflux MSF transporter	0	1
*cflA*	Drug efflux MSF transporter Bcr/CflA family	0	1

^1^*sul2* was found in strains NIPH 478, NIPH 715, TG19636; ^2^ Genes *aph*(3″)-Ib and *aph*(6)-I were found in strain TG19636.

**Table 6:** Mobile elements in environmental strains of *A. lwoffii*.

Strain	ED23-35	ED45-23	ED9-5A	VS15	EK30A	GC2	ZS207 ^1^	M2a ^1^
Total number of copies of IS elements	78	70	107	98	133	25	86	205
Number of IS elements	25	29	34	33	39	23	40	49
Number of families of IS elements	9	9	11	11	11	10	15	13
Composite transposons	2	4	0	3	4	0	0	2
Assembly quality (number of scaffolds) ^2^	C/P	C/P	C/P	C/P	C/P	285	C/P	277

^1^ data from reference [32]; ^2^ C/P, complete chromosome and plasmids.

**Table 7:** Mobile elements in clinical strains of *A. lwoffii*.

Strain	SH145	NIPH 715	CIP 70.31	NIPH 478	NIPH 512	TG19636
Total number of copies of IS elements	7	90	115	19	57	19
Number of IS elements	7	30	31	8	18	19
Number of families of IS elements	4	19	11	3	9	10
Composite transposons	0	0	0	0	0	0
Assembly quality (number of scaffolds)	76	26	12	9	12	245

**Table 8:** Intact prophages identified in the genomes of permafrost strains of *A. lwoffii*.

Strain	Phage	Length, (kb)	Position	Location	Similar Prophages (Coverage ^1^/Identity, %)
ED23-35	ED23-35-3	34.1	1709101-1743234	Chromosome	-
	ED23-35-6	20	2260258-2280259	Chromosome	EK30A-7 (30/93.4)
ED45-23	ED45-23-1	33.6	1543115-1576799	Chromosome	-
	ED45-23-4	43.7	2081979-2125746	Chromosome	-
	ED45-23-7	50.4	3208722-3259176	Chromosome	VS15-6 (45/91.4) EK30A-8 (30/94.3)
ED9-5A	ED9-5A-1	37.8	994113-1031993	Chromosome	-
	ED9-5A-3	39	2063965-2103060	Chromosome	-
	ED9-5A-6	18.9	972-19932	pALWED 3.6	ED23-35-6 (36/93.8)
VS15	VS15-6	49.6	2753607-2803257	Chromosome	ED45-23-7 (46/91.4)
EK30A	EK30A-7	36.6	1504782-1541418	Chromosome	-
	EK30A-8	24.5	2890359-2914858	Chromosome	ED45-23-7 (55/94.3) VS15-6 (38/95.5)

^1^ Only similarities covering more than 30% of the prophage length.

## Data Availability

All sequences associated with this work have been deposited at the National Center for Biotechnology Information under BioProject ID: PRJNA325719.

## Supplementary Materials

The following are available online at https://www.mdpi.com/article/10.3390/biology10090871/s1, Table S1: Sequencing statistics, Table S2: Pairwise nucleotide sequence identity (%) between the genomes of *A. lwoffii* strains, Table S3: List of genes present in all 8 environmental strains but missing in at least one of 6 clinical strains, Table S4: List of genes present in all 6 clinical strains but missing in at least one of 8 environmental strains, Table S5: Distribution of IS elements in the genomes of clinical and environmental strains of *A. lwoffii*, Table S6: Composite transposons in the genomes of *A. lwoffii*., Table S7: Resistance genes on plasmids and chromosomes of contemporary strains of *A. lwoffii*.
