# Supplementary material for: Genome Analysis of Acinetobacter lwoffii Strains Isolated from Permafrost Soils Aged from 15 Thousand to 1.8 Million Years Revealed Their Close Relationships with Present-Day Environmental and Clinical Isolates
Source: Biology (Basel). 2021 Sep 4;10(9):871. doi: 10.3390/biology10090871 (PMC8472584; doi:10.3390/biology10090871)
Supplement: Supplementary file 1 [file biology-10-00871-s001.zip › Supplemental Table S6.pdf]

Supplementary Table S6. Composite transposons in the genomes of *A. lwoffii*.

1) Strain ED23-35
Size of composite transposon: 8163 bp

Description	Coordinates		Orientation
	start	stop	
ISAba14	2660338	2661619	>
Rep_3. Plasmid replication. RepB (partial)	2661982	2661590	<
hypothetical protein	2662787	2663095	>
hypothetical protein	2663715	2663245	<
hypothetical protein	2664335	2663865	<
MobA	2665285	2664332	<
hypothetical protein	2665500	2665285	<
MobC	2665694	2665963	>
hypothetical protein	2666546	2665974	<
Rep_3. Plasmid replication. RepB (partial)	2667026	2666634	<
ISAba14	2667219	2668500	>

2) Strain ED23-35
Size of composite transposon: 10174 bp

Description	Coordinates		Orientation
	start	stop	
ISAba11	3087317	3088417	>
Uncharacterized sodium-dependent transporter YocS	3089441	3088494	<
Nicotinamidase (EC 3.5.1.19)	3090199	3089570	<
4-hydroxybenzoate transporter	3091713	3090508	<
4-hydroxy-tetrahydrodipicolinate synthase (EC 4.3.3.7)	3092438	3093337	>
putative lipoprotein-34 precursor (NlpB)	3093352	3093957	>
Phosphoribosylaminoimidazole-succinocarboxamide synthase (EC 6.3.2.6)	3093995	3094714	>
Replication-associated recombination protein RarA	3096292	3095024	<
ISAba11	3096387	3097487	>

3) Strain ED45-23
Size of composite transposon: 3410 bp

Description	Coordinates		Orientation
	start	stop	
ISAba1	2471751	2472930	<
4-hydroxybenzoate polyprenyltransferase (EC 2.5.1.39)	2472965	2473843	>
ISAba1	2473981	2475160	<

Note that identical composite transposon is present in *Acinetobacter baumannii* EH

4) Strain ED45-23
Size of composite transposon: 8243bp.

Description	Coordinates		Orientation
	start	stop	
ISAba125	2470396	2471482	>
ISAba1	2471751	2472930	<
4-hydroxybenzoate polyprenyltransferase (EC 2.5.1.39)	2472965	2473843	>
ISAba1	2473981	2475160	<
Alkyl sulfatase and related hydrolases, MBL-fold metallo-hydrolase superfamily	2477207	2475252	<
ΔISAlw27	2477228	2477519	>
ISAba125	2477552	2478638	>

5) Strain ED45-23
Size of composite transposon: 10959 bp

Description	Coordinates		Orientation
	start	stop	
ISAlw9	1325268	1326659	<
16S rRNA (cytosine(1402)-N(4))-methyltransferase (EC 2.1.1.199)	1326811	1327722	>
Cell division protein FtsL	1327733	1328065	>
Cell division protein FtsI [Peptidoglycan synthetase] (EC 2.4.1.129)	1328081	1329904	>
UDP-N-acetylmuramoylalanyl-D-glutamate--2,6-diaminopimelate ligase (EC 6.3.2.13)	1329916	1331412	>
UDP-N-acetylmuramoyl-tripeptide--D-alanyl-D-alanine ligase (EC 6.3.2.10)	1331422	1332825	>
Phospho-N-acetylmuramoyl-pentapeptide-transferase (EC 2.7.8.13)	1332826	1333944	>
Ribosomal RNA large subunit methyltransferase A (EC 2.1.1.51)	1333999	1334802	>
ISAlw9	1334835	1336226	<

Note that similar composite transposon (90% nucleotide sequence identity) is present in *Acinetobacter lwoffii* strain FDAARGOS 1393.

6) Strain ED45-23
Size of composite transposon: 2618 bp

Description	Coordinates		Orientation
	start	stop	
ISAba125	2634122	2635208	<
type III restriction enzyme, res subunit	2635283	2635669	>
ISAba125	2635653	2636739	<

7) Strain VS15
Size of composite transposon: 2618 bp.
Two identical copies located in the chromosome and in plasmid pALWVS1.4.

Description	Coordinates		Orientation
	start	stop	
ISAba1	1976802	1977981	<
Multidrug resistance transporter, Bcr/CflA family	1979256	1978060	<
ISAba1	1980072	1981251	<

8) Strain VS15
Size of composite transposon: 10956 bp

Description	Coordinates		Orientation
	start	stop	
ISAlw10	1080659	1082047	<
Ribosomal RNA large subunit methyltransferase A (EC 2.1.1.51)	1082882	1082079	<
Phospho-N-acetylmuramoyl-pentapeptide-transferase (EC 2.7.8.13)	1084055	1082937	<
UDP-N-acetylmuramoyl-tripeptide--D-alanyl-D-alanine ligase (EC 6.3.2.10)	1085459	1084056	<
UDP-N-acetylmuramoylalanyl-D-glutamate--2,6-diaminopimelate ligase (EC 6.3.2.13)	1086970	1085474	<
Cell division protein FtsI [Peptidoglycan synthetase] (EC 2.4.1.129)	1088805	1086982	<
Cell division protein FtsL	1089153	1088821	<
16S rRNA (cytosine(1402)-N(4))-methyltransferase (EC 2.1.1.199)	1090075	1089164	<
ISAlw10	1090226	1091614	<

9) Strain EK30A
Size of composite transposon: 3524 bp.

Description	Coordinates		Orientation
	start	stop	
ISAlw5	367311	368619	<
hypothetical protein	368644	369528	>
ISAlw5	369526	370834	<

10) Strain EK30A
Size of composite transposon: 10861 bp.

Description	Coordinates		Orientation
	start	stop	
ISAba1	2870992	2872171	>
hypothetical protein	2872712	2873182	>
hypothetical protein; putative exported protein	2874354	2873371	>
fimbrial adhesin precursor	2875527	2874514	>

outer membrane usher protein precursor	2878076	2875524	>
P pilus assembly protein, chaperone PapD	2878869	2878138	>
Fimbrial protein	2879489	2878956	>
ISAba1	2880673	2881852	>

Note that identical composite transposon is present in *Acinetobacter lwoffii* strain FDAARGOS_552 (plasmid unnamed 1)

11) Strain EK30A
Size of composite transposon: 8022 bp.

Description	Coordinates		Orientation
	start	stop	
ISAlw9	2899792	2901184	<
hypothetical protein	2901547	2901206	<
hypothetical protein	2902755	2902012	<
hypothetical protein	2902928	2902755	<
hypothetical protein	2903360	2902938	<
Phage protein	2903823	2903335	<
Transglycosylase-associated protein	2904288	2903824	<
hypothetical protein	2904568	2904359	<
hypothetical protein	2905277	2904636	<
hypothetical protein	2905674	2905237	<
hypothetical protein	2906180	2906067	<
hypothetical protein	2906678	2906824	>
hypothetical protein	2907310	2907128	<
ISAlw9	2907783	2909172	<

12) Strain EK30A
Size of composite transposon: 5481 bp.

Description	Coordinates		Orientation
	start	stop	
ISAba125	2481090	2482174	>
IcmF-related protein	2484010	2482352	<
Esterase ybfF (EC 3.1.-.-)	2484883	2484122	<
ISAba125	2485484	2486570	>
