# Supplementary material for: Genome Analysis of Acinetobacter lwoffii Strains Isolated from Permafrost Soils Aged from 15 Thousand to 1.8 Million Years Revealed Their Close Relationships with Present-Day Environmental and Clinical Isolates
Source: Biology (Basel). 2021 Sep 4;10(9):871. doi: 10.3390/biology10090871 (PMC8472584; doi:10.3390/biology10090871)
Supplement: Supplementary file 1 [file biology-10-00871-s001.zip › Supplemental Table S7.pdf]

Supplementary Table S7. Resistance genes on plasmids and chromosomes of contemporary strains of *A. lwoffii*

Strain	Source	Chromosome GenBank Acc. No. (resistance genes*)	Plasmids** (GenBank Acc. No.)	Resistance genes (plasmid)
12CE1	Prawn	CP059081.1 (*tel, cop, ars, ohr*)	**pR4WN_12CE1** (MT742180)	*sul, aadA, qacE*
FDAARGOS_551	Homo sapiens (clinical)	CP054822.1 (*tel, cop, ars, ohr*)	**Unnamed1** (CP054821.1)	*ars, cop, ohr****
			Unnamed2 (CP054824.1)	
			Unnamed3 (CP054823.1)	
			Unnamed4 (CP054825.1)	*chr*
			Unnamed5 (CP054826.1)	
FDAARGOS_552	Homo sapiens (clinical)	CP046296.1	**Unnamed1** (CP046295.1)	*czc, ars, cop*
			Unnamed2 (CP046297.1)	
			Unnamed3 (CP046298.1)	
			Unnamed4 (CP046299.1)	
FDAARGOS_557	Homo sapiens (clinical)	CP054803.1 (*tel, cop, ars, ohr*)	Unnamed1 (NZ_CP054806.1)	
			Unnamed2 (CP054805.1)	
			**Unnamed3** (CP054804.1)	*ars, cop, ohr*
FDAARGOS_620	Homo sapiens (clinical)	NZ_JAAXYZ010000003.1 (*cop, ars, ohr*)	**Unnamed1** (NZ_JAAXYZ010000001.1)	*ars, cop, ohr, czc*
			Unnamed2 (NZ_JAAXYZ010000002)	*chr*
			Unnamed2 (NZ_JAAXYZ010000005.1)	
M2a	Honey	-	**pAVAci14** (MK978162.1)	
			pAVAci84 (MK944320.1)	
			pAVAci94 (MK978163.1)	
			pAVAci98 (MK993303.1)	*cop*

			pAVAci115 (MK993300.1)	
			pAVAci116 (MK978161.1)	
			pAVAci117 (MK978159.1)	
			pAVAci119 (MK978160.1)	
			pAVAci127 (MK993301.1)	
			pAVAci130 (MK944319.1)	
			pAVAci144 (MK944317.1)	
			pAVAci145 (MK944318.1)	
			pAVAci147 (MK944321.1)	
			pAVAci167 (MK993302.1)	
			pAVAci176 (MK944322.1)	
FDAARGOS 1393	missing info	CP077336.1 (*tel, cop, ars, ohr*)	**Unnamed1** (CP077337.1)	
			**Unnamed2** (CP077338.1)	*ars, cop*
			**Unnamed3** (CP077339.1)	*chr, ars, mer*
			Unnamed4 (CP077340.1)	
			Unnamed5 (CP077341.1)	
FDAARGOS 1394	missing info	CP077369.1 (*tel, cop, ars, ohr*)	**Unnamed1** (CP077370.1)	*cop*
			**Unnamed2** (CP077371.1)	
			Unnamed3 (CP077372.1)	
			Unnamed4 (CP077373.1)	
			Unnamed5 (CP077374.1)	
			Unnamed6 (CP077375.1)	
			Unnamed7 (CP077376.1)	
			Unnamed8 (CP077377)	
ZS207	Gold mine	CP019143.2	**pmZS** (CP019144)	*ars*
			pZS-1	

			(CP043941)	
			pZS-2 (CP019152)	
			pZS-3 (CP019145)	
			pZS-4 (CP019147)	
			pZS-5 (CP019146)	
			pZS-6 (CP019151)	
			pZS-7 (CP019148)	
			pZS-8 (CP019149)	
			pZS-9 (CP019150)	
			pZS-11 (NZ_CP019150)	
			pZS-13 (NZ_CP019151)	
			pZS-20 (NZ_CP019152)	
SU1904	Bile	-	**pSU1904NDM.1** (LC537594.1)	*aphA6, bla*$_{\text{NDM-1}}$
WJ10621	Homo sapiens (clinical)	NZ_CM001194.1	**pNDM-BJ01** (JQ001791.1)	*bla*$_{\text{NDM-1}}$, *aphA6, ble*
WJ10659	Homo sapiens (clinical)	-	**pNDM-BJ02** (JQ060896.1)	*bla*$_{\text{NDM-1}}$, *aphA6, ble*
Iz4b	Homo sapiens	-	**pNDM-Iz4b** (NC_025000.1)	*bla*$_{\text{NDM-1}}$, *aphA6, ble*
JN49-1	Homo sapiens (feces)	-	**pNDM-JN01** (KM210086.1)	*bla*$_{\text{NDM-1}}$, *aphA6, ble*
SGC-HZ9	Chicken (cloacal swab)		pAL-01 (JN616388)	*bla*$_{\text{NDM-1}}$, *aphA6*
ABZ78	Clinical isolate	-	pABZ78 (JQ739158)	*ble*

* resistance genes in addition to the genes *cat*, *bla*$_{\text{OXA-134}}$, *macAB* present in the chromosomes of all the studied strains

** plasmids larger than 40 kb are highlighted in bold

Resistance genes: *ohr* – resistance to organic hydroperoxides, *tel* – to tellurium, *aphA6*- to amikacin, *ble* – to bleomycin, *aadA* – to streptomycin / spectinomycin, *qacE* – to quartenary ammonium compounds, sul – to sulfonamide, *nreB* – to Ni, *bla*$_{\text{NDM-1}}$ – broad-spectrum beta-lactamases., *czc* – to Co/Zn/Cd, *cop* – to Cu, *chr* – to Cr, *ars* – to As; *mer* – mercury resistance operon.
